# Balanced multi-electrolyte solution versus normal saline for fluid therapy in aneurysmal subarachnoid hemorrhage: an influence on fluid and electrolytes balance and outcome

**DOI:** 10.3389/fmed.2025.1708812

**Published:** 2025-11-26

**Authors:** Yu-Qing Duan, Ying Tian, Shuya Wang, Shan-Shan Xu, Ming-Yue Miao, Ran Gao, Rui Su, Mengxue Hou, Yuqing Chen, Zimeng Xu, Linlin Zhang, Jian-Xin Zhou

**Affiliations:** 1 Department of Critical Care Medicine, Beijing Tiantan Hospital, Capital Medical University, Beijing, China; 2 Clinical and Research Center on Acute Lung Injury, Emergency and Critical Care Medical Center, Beijing Shijitan Hospital, Capital Medical University, Beijing, China; 3 Department of Neurocritical Care, Beijing Anzhen Hospital, Capital Medical University, Beijing, China

**Keywords:** aneurysmal subarachnoid hemorrhage, normal saline, balanced multi-electrolyte solution, isotonic solutions, hyperchloremia

## Abstract

**Introduction:**

Isotonic crystalloids are commonly used for maintaining fluid balance and cerebral perfusion pressure in critical care patients with aneurysmal subarachnoid hemorrhage (aSAH). However, the relatively high concentration of chloride in normal saline (NS) might lead to hyperchloremia or acute kidney injury, comparing with multi-electrolyte solutions (BMES). The aim of the study is to compare the incidence of hyperchloremia in aSAH patients and provide feasibility and safety research for further study.

**Methods:**

This is a pilot study of a single center, randomized, controlled trail. Patients were enrolled randomly to receive BMES or NS for 3 days of ICU stay.

**Results:**

Overall, 87 patients were randomized to receive BMES or NS, 60 patients (30 in each group) were enrolled for final analysis. Within 3 days of randomization, hyperchloremia occurred in 18/30 (60%) patients in the BMES group and 23/30 (76.7%) in the NS group (*p* = 0.165, relative risk 0.58, 95% CI 0.27–1.28). Incidence of hyperchloremia (BMES 36.7% vs. NS 63.3%, *p* = 0.039) and hyperchloremic acidosis (BMES 36.7% vs. NS 63.3%, *p* = 0.039) were decreased on trial day 1. There were no differences on bicarbonate, anion gap, serum creatinine, incidence of acute kidney injury, or length of hospital stay between groups.

**Discussion:**

For patients with aSAH, the use of BMES did not result in a lower risk of hyperchloremia, and also did not increase the incidence of hyponatremia or intracranial hypertension over NS, which warrants further research.

## 1 Introduction

Aneurysmal subarachnoid hemorrhage (aSAH) is a significant public health threat among neurological diseases, with a global incidence of 6.1 per 100,000 person-years (1). The 30 days mortality of SAH is close to 30% (2) and the incidence of moderate to severe disability is over 10% (3). A major cause of poor prognosis in SAH patients is the occurrence of delayed cerebral ischemia, mainly caused by insufficient blood supply due to cerebral vasospasm. Current guidelines (4) are inclined to maintain adequate blood pressure and cerebral perfusion pressure in order to improve cerebral blood flow. For which, a large amount of fluid is often needed for blood volume expanding in clinical operations.

Normal saline (NS) was commonly used to prevent hyponatremia, which is associated with poorer outcomes in aSAH patients (5–7). However, given the evidence of improved inflammatory markers and the fact that NS delivers supra-physiological chloride load (154 mmol/L) in other diseases (8, 9), balanced multi-electrolyte solutions (BMES) may be the preferred crystalloid over NS. Excess chloride load brought by NS can cause hyperchloremia, which has been linked to an increased risk of hyperchloremic acidosis, kidney injury, and poor outcomes (10–13).

Despite the potential benefits of chloride restriction, recent clinical trials in large cohorts of critically ill patients have failed to show any improvement in mortality, which may be attributed to the highly heterogeneous populations that do not often receive large amounts of fluids (14, 15). Interestingly, SMART trial (13) showed that balanced solution infusions were associated with better clinical benefits in neurologic units. While the SALT trial (16) results indicated that the clinical impact of BMES versus NS may be volume-dependent. SAH patients with large fluid amount requirements may benefit more from it. Due to a lack of evidence, the choice of a balanced solution or NS in aSAH patients remains controversial, and whether BMES is a good candidate is unknown.

Therefore, we designed this pilot randomized trial aiming to compare the effect of BMES versus NS on the occurrence of hyperchloremia within 72 h in patients with aSAH. A secondary aim was to provide data for the design and power of a large-scale, multicenter, randomized controlled trial.

## 2 Methods

### 2.1 Trial design

This is a single-center, parallel, randomized controlled pilot trial conducted in Beijing Tiantan Hospital, Capital Medical University, from November 1, 2021 (the first enrollment) to May 31, 2023 (the last enrollment). All investigators were blinded from the randomization results. Study was approved by the ethics committees of Beijing Tiantan Hospital (KY2023-035-02). Written informed consent was obtained from all patients or their next of kin. All authors had access to the study data and reviewed and approved the final manuscript. Our trial was registered on clinicaltrails.gov (NCT06076590).

### 2.2 Trial participants

The inclusion criteria for this trial were the presence of aSAH, with an expected intensive care unit (ICU) stay of more than 24 h. Patients were excluded if they were under the age of 18, pregnant, refused to sign the informed consent, had a history of severe cardiovascular, respiratory, kidney, liver, blood, or immune diseases, or had received renal replacement therapy prior to randomization. Patients were also excluded if they present with nitrogenemia, hypokalemia, hypocalcemia. The follow-up was completed on May 28th, 2023.

### 2.3 Randomization and blinding

All study subjects were randomized to receive BMES or NS after enrollment. The randomization sequence was generated by computer. A research coordinator sealed the randomization assignments in sequentially numbered opaque envelopes. When a study participant consented and was enrolled, the same research coordinator opened envelopes sequentially. Study investigators, phlebotomists, and biostatisticians responsible for data analyses were all blinded to patient allocation.

### 2.4 Trial interventions

Patients allocated to the NS group received 0.9% saline (154 mmol/L sodium and 154 mmol/L chloride) or 0.9% sodium chloride/ 5% dextrose injection as maintenance fluids when isotonic intravenous fluid administration was ordered by the treating physician for three consecutive days after enrollment or until discharge or death, whichever happened first. Patients allocated to the BMES group received Multiple Electrolytes injection II (Sterofundin ISOTM, B. Braun, Germany, see electrolyte contents in Supplementary Table S1) when isotonic intravenous fluid administration was ordered for the same period.

Mannitol is given priority during this study if osmotic therapy is required for treating cerebral edema after aneurysm occlusion surgery. Hypertonic saline (3% sodium chloride) could only be used when the application of mannitol is ineffective (intracranial pressure over 20 mmHg after 10 min of application), and the dosage should be recorded. Concentrated sodium chloride (10% sodium chloride) should only be administered when blood sodium is below 130 mmol/L, and the dosage should be recorded. Glucose injection could be administered in patients, sodium level over 150 mmol/L. Patients were withdrawn from the study when blood sodium increased beyond 160 mmol/L. Other treatment options are determined by the treatment clinician.

### 2.5 Data collection

Data were collected from the electronic medical record and nursing record using a standardized case report form. All imaging results were reviewed by at least two experienced radiologists. Two independent investigators who were unaware of the study allocation evaluated the outcome measures. If two investigators disagreed on any study outcome assessed, a third investigator was consulted, and majority decisions were made.

### 2.6 Trial endpoints

The primary endpoint was the development of hyperchloremia during the first 3 days after enrollment. Chloride levels were measured from the morning blood samples during the first 3 days after randomization, with the day of randomization being labeled day 0, the next day day 1, and the following 2 days day 2 & day 3. The automated biochemistry analyzer in our central laboratory was used to measure all chloride levels according to standard protocols. Hyperchloremia is defined as a serum chloride concentration of more than 108 mmol/L, which exceeds the normal range of our hospital’s biochemical laboratory.

Clinical secondary outcome endpoints include the incidence of hyperchloremia each day, new-onset hyperchloremia, hyperchloric acidosis, and changes in intracranial pressure (ICP) during the treatment period, new-onset or deteriorated acute kidney injury (AKI), requirement of renal replacement therapy (RRT), duration of mechanical ventilation, length of ICU stay, length of hospital stay, hospitalization costs, and Glasgow outcome scale (GOS) score at ICU discharge. Acute kidney injury was diagnosed according to Kidney Disease Improving Global Outcomes (KDIGO) consensus criteria (17).

Laboratory secondary endpoints include daily serum chloride, sodium, potassium, calcium, blood urea nitrogen (BUN), creatinine and arterial pH, partial pressure of carbon dioxide (PCO_2_), anion gap (AG), bicarbonate (HCO_3_^−^), base excess (BE), strong ion difference (SID), osmotic pressure (Osm) and lactate (Lac) for the first 3 days of randomization.

### 2.7 Statistical analysis

A formal sample size calculation was not performed because this was a pilot study. A pragmatic sample size of 30 participants was chosen because it was thought to provide meaningful safety information while also minimizing potential harm exposure. All analyses were conducted based on the per-protocol population. We present continuous data as median (interquartile range, IQR) or mean (standard deviation, SD), depending on the normality. The normality of data was determined by Kolmogorov–Smirnov tests. Comparisons of continuous data were conducted using the Mann–Whitney U test or Student’s t-test as appropriate. Categorical data were assessed with Fisher’s exact test or chi-square test, as indicated. Risk ratios (RR) with 95% confidence intervals (CIs) were calculated for categorical variables. The repeated measures analysis of variance was used to test the between-group differences in repeated measurements. We considered a two-sided *p*-value of less than 0.05 to be statistically significant. All statistical analyses were done in the SPSS 26.0 software (SPSS Inc., Chicago, IL, USA).

## 3 Results

### 3.1 Trial recruitment and baseline characteristics

**Figure 1:**
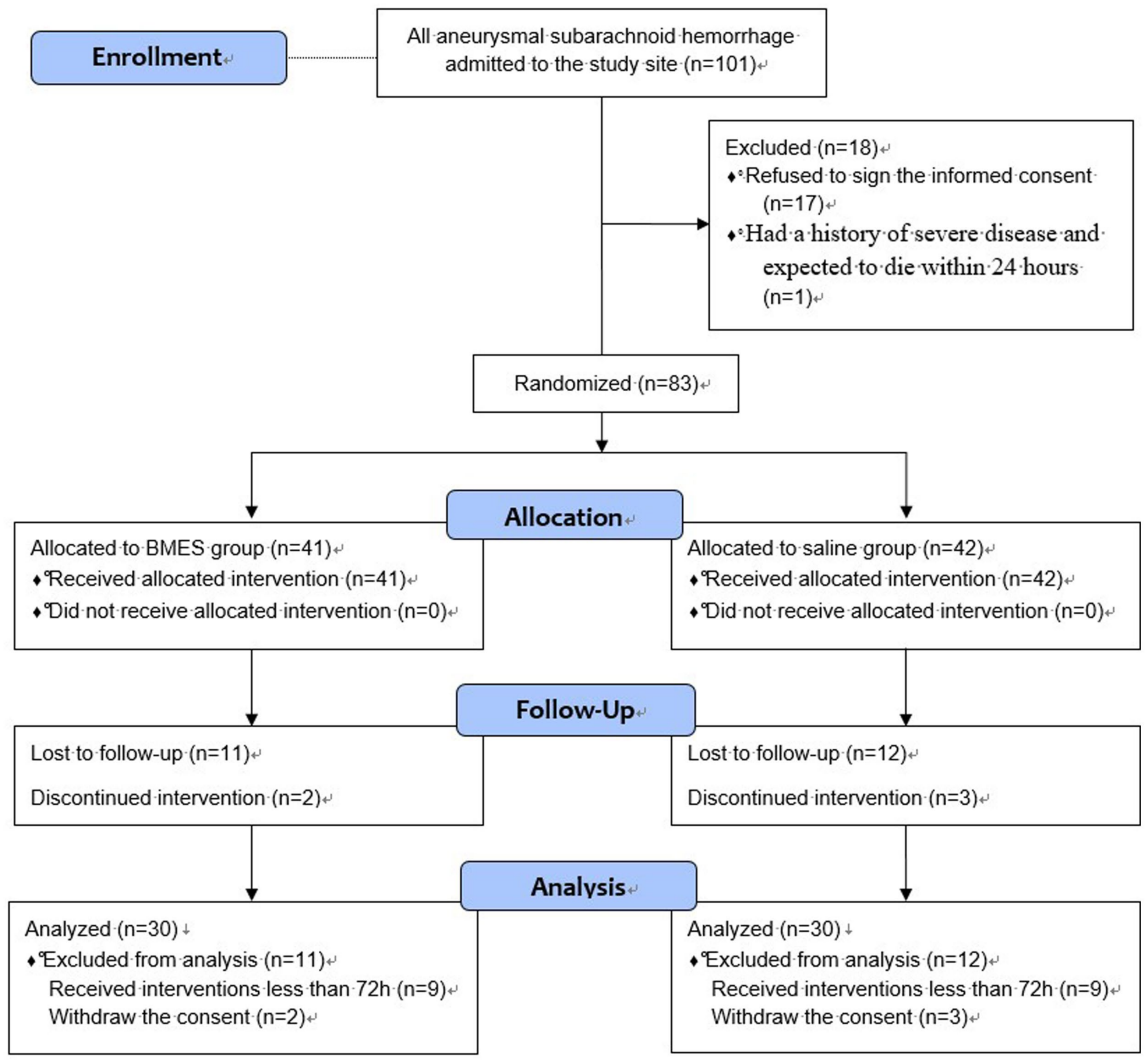
Flow Diagram.

During the 19-month trial period, 101 patients with aSAH were screened for eligibility. In total, 83 patients were randomly assigned to receive either BMES or NS. During the follow-up period, 9 patients in each group received interventions lasting less than 72 h and lost follow-up. In addition, two patients in the BMES group and three patients in the NS group withdrew their informed consent. Finally, 30 patients from each group were included in further analyses (Figure 1).

Overall, the median age was 56 years (IQR 50–66), and the percentage of male gender was 56.7% (34/60). At enrollment, approximately all of the study patients (57/60, 95.0%) received emergency surgery, and the median surgery duration was 4.0 h (IQR 3.5–4.8). Baseline electrolyte, acid–base parameters, and other laboratory parameters were shown in Supplementary Table S2. There were no differences in baseline characteristics between the two groups (Table 1; Supplementary Table S2).

**Table 1:** Baseline characteristics of the study subjects.

Characteristics	BMES group (*n* = 30)	NS group (*n* = 30)	*p*
Age, yr., median (IQR)	57 (48, 66)	55 (49, 65)	0.679
Gender, male, n (%)	17 (56.7)	17 (56.7)	1.000
Height, cm, median (IQR)	169 (160, 170)	165 (158, 172)	0.976
Weight, kg, median (IQR)	69 (61, 75)	66 (63, 81)	0.324
Hypertension, n (%)	19 (63.3)	20 (66.7)	0.787
Emergency surgery, n (%)	28 (93.3)	29 (96.7)	0.554
Duration of surgery, hr., median (IQR)	3.9 (3.3, 4.7)	4.2 (3.7, 4.9)	0.129
ICP, mmHg, median(IQR)	13 (10, 18)	16 (9, 16)	0.916
Hunt-Hess grade, median (IQR)	3 (2, 3)	3 (2, 3)	0.719
Modified Fisher grade, median (IQR)	3 (2, 3)	3 (2, 4)	0.294
Baseline creatinine, μmol/L, median (IQR)	54 (47, 61)	57 (43, 71)	0.580
GCS score, median (IQR)	15 (6, 15)	8 (7, 15)	0.181
SOFA score, median (IQR)	2 (1, 5)	4 (2, 6)	0.063
APACHE II score, median (IQR)	10 (7, 15)	12 (8, 16)	0.441

BMES denotes balanced multielectrolyte solutions, NS denotes normal saline, IQR denotes interquartile range, GCS denotes glasgow coma scale, SOFA denotes sequential organ failure assessment score, APACHE II denotes acute physiology and chronic health evaluation II.

### 3.2 Fluid administered

All study patients received the assigned fluid as the preferred resuscitation and overall hydration intravenous fluid for 72 h. There was no difference in overall fluid input during the study period, while BMES group received 4,000 mL infusion of study fluid and NS group received 4,150 mL (Table 2). The most common reason for the prescription of NS was to dissolve intravenous drugs as required by the drug labeling, and the volume was strictly limited. We also estimated the volume of osmotic therapy including mannitol and hypertonic saline, which both might has significant influence on electrolytes levels and no statistical difference was found. Volumes of other intravenous fluids, including dextrose solution, parenteral nutrition, mannitol, and blood products administered during the first three trial days, are shown in Supplementary Table S3. Difference in potassium chloride intake may be due to the allowance of 0.9% sodium chloride/ 5% dextrose injection as study fluid in the NS group which resulted in more insulin as well as potassium solution.

**Table 2:** Fluid intakes during intervention period.

Fluids	BMES group (*n* = 30)	NS group (*n* = 30)	*p*
Fluid input, ml, median (IQR)	10,225 (9,034, 12,525)	11,039 (10,088, 12,125)	0.137
BMES, ml, median (IQR)	4,000 (3,000, 4,625)	0 (0, 0)	<0.001
NS, ml, median (IQR)	500 (0, 1,275)	4,150 (3,425, 5,500)	<0.001
Mannitol, ml, median (IQR)	1,125 (0, 2063)	1,500 (1,000, 2,250)	0.244
Diet intake^a^, ml, median (IQR)	2,935 (2,333, 4,180)	3,150 (2,403, 4,500)	0.641
Compatibility solution (NS)^b^, ml, median (IQR)	1,348 (850, 2009)	1,525 (1,088, 2,209)	0.198
Compatibility solution (5%Glucose)^b^, ml, median (IQR)	0 (0, 0)	0 (0, 263)	0.080
15% Potassium chloride solution, ml, median (IQR)	30 (15, 86)	103 (70, 120)	<0.001
Other drugs^c^, ml, median (IQR)	140 (11, 225)	157 (102, 227)	0.249

^a^The Diet intake includes nasal-fed intestinal nutrient solution and mouth-feeding liquid; ^b^The compatibility solution refers to the liquid used to dissolve and dilute drugs; ^c^Other drugs include various intravenous drugs that do not need to be dissolved, such as propofol and nicodipine, etc. BMES denotes balanced multielectrolyte solutions, NS denotes normal saline, IQR denotes interquartile range.

### 3.3 Primary outcome and secondary outcomes

**Figure 2:**
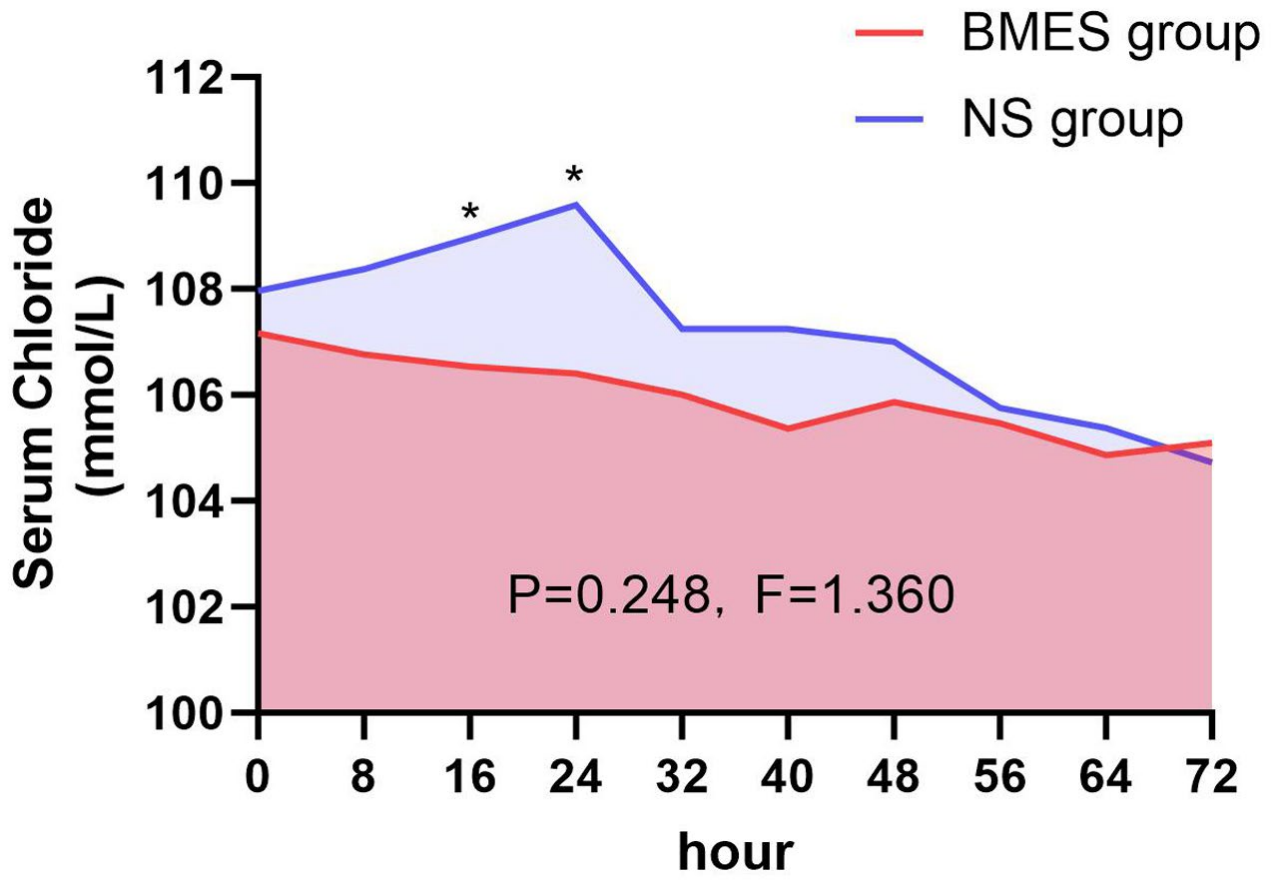
Serum chloride concentration during treatment period. Mean serum chloride levels in the BMES and NS groups are presented at 8-hour intervals. Statistically differences between groups were observed at 16 and 24 hour post-enrollment, whereas no significant difference was detected over the entire 72-hour intervention period.

On trial day three, all the study patients had their serum chloride levels measured in the morning. As shown in Table 3, hyperchloremia occurred in 18/30 (60%) patients in the BMES group and 23/30 (76.7%) in the NS group (*p* = 0.165, relative risk 0.58, 95%CI 0.27–1.28) within 72 h after randomization. The incidence of hyperchloremia were decreased on day 1 and day 2 (Table 3). The daily serum chloride concentrations are shown in Figure 2. During the first three-day after randomization, there was no difference in serum chloride concentration between the two groups (*F* = 1.36, *p* = 0.248). However, there was a significant difference at 16 h after randomization, with an average chloride level of 106.5 mmol/L in BMES group and 109.0 mmol/L in NS group (*p* = 0.029). The gap further widened to 106.4 mmol/L in BMES and 109.6 mmol/L in NS group at 24 h (*p* = 0.017, Supplementary Table S4).

**Table 3:** Primary and secondary outcomes.

Outcomes	BMES group (*n* = 30)	NS group (*n* = 30)	Relative risk (95%CI)	*p*
Hyperchloremia within 72 h, *n* (%)	18 (60.0)	23 (76.7)	0.58 (0.27, 1.28)	0.165
Hyperchloremia at day 0, *n* (%)	13 (43.3)	15 (50.0)	0.88 (0.55, 1.42)	0.605
Hyperchloremia at day 1, *n* (%)	11 (36.7)	19 (63.3)	0.58 (0.34, 0.997)	0.039
Hyperchloremia at day 2, *n* (%)	8 (26.7)	16 (53.3)	0.50 (0.25, 0.99)	0.035
Hyperchloremia at day 3, *n* (%)	6 (20.0)	11 (36.7)	0.79 (0.57, 1.10)	0.152
New onset Hyperchloremia, *n* (%)	5 (16.7)	8 (26.7)	0.88 (0.67, 1.15)	0.347
Hyperchloric acidosis within 72 h, *n* (%)	18 (60.0)	23 (76.7)	0.58 (0.27, 1.28)	0.165
Hyperchloric acidosis at day 0, *n* (%)	12 (40.0)	15 (50.0)	0.83 (0.53, 1.32)	0.436
Hyperchloric acidosis at day 1, *n* (%)	11 (36.7)	19 (63.3)	0.58 (0.34, 0.997)	0.039
Hyperchloric acidosis at day 2, *n* (%)	7 (23.3)	10 (33.3)	0.87 (0.63, 1.20)	0.390
Hyperchloric acidosis at day 3, *n* (%)	5 (16.7)	9 (30.0)	0.84 (0.63, 1.12)	0.222
New-onset or deteriorated AKI	1 (3.3)	2 (6.7)	0.97 (0.86, 1.09)	0.554
Requirement of CRRT, *n* (%)	0 (0)	0 (0)	-	1.000

BMES denotes balanced multielectrolyte solutions, NS denotes normal saline, CRRT denotes continuous renal replacement therapy.

**Figure 3:**
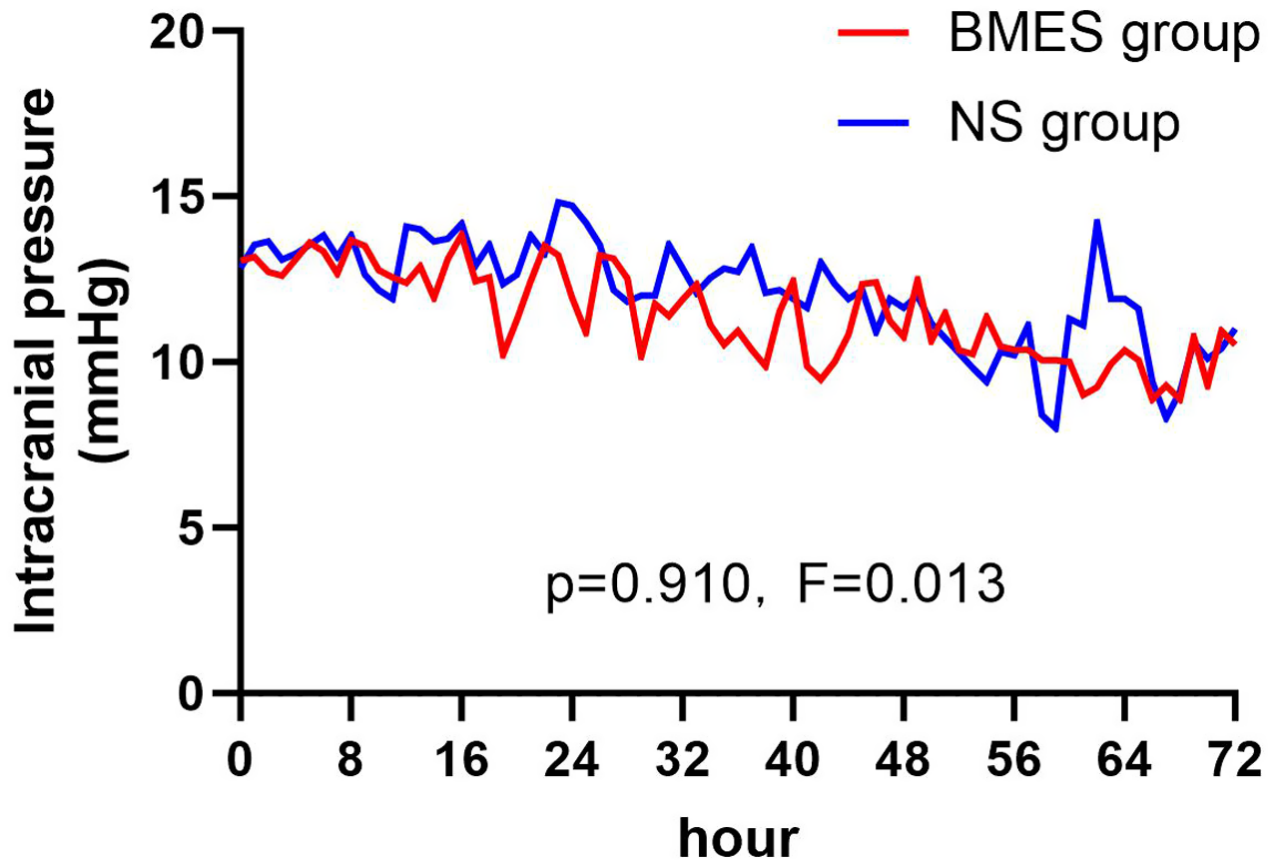
Intracranial pressure during treatment period. Mean intracranial pressure in the BMES and NS groups are presented at 8-hour intervals.

With regard to the secondary endpoints, patients who received the BMES for their initial fluid therapy had less hyperchloric acidosis on day 1 (36.7% [11/30] versus 63.3% [19/30], *p* = 0.039). There were no differences between groups with regards to other secondary outcomes including new-onset or deteriorated acute kidney injury (AKI), requirement of renal replacement therapy (RRT) (Table 3). Inflammatory parameters, duration of mechanical ventilation, length of ICU stay, length of hospital stay, hospitalization costs, and GOS score at ICU discharge (Tables 4, 5). The daily ICPs are shown in Figure 3. There are no differences in daily ICP between the two groups during the first three-day after randomization (*F* = 0.013, *p* = 0.91).

**Table 4:** Inflammatory parameters.

Inflammatory parameters	BMES group (*n* = 30)	NS group (*n* = 30)	*p*
WBC, 10^9^/L, median (IQR)	18.0 (15.1, 21.3)	18.4 (15.2, 22.7)	0.947
NEUT, 10^9^/L, median (IQR)	15.6 (13.4, 18.4)	15.8 (11.4, 19.9)	0.923
PLT, 10^9^/L, median (IQR)	332 (268, 406)	385 (300, 492)	0.107
CRP, mg/L, median (IQR)	112.2 (70.0, 173.2)	114.6 (55.3, 207.4)	0.976
IL-6, ng/L, median (IQR)	38.4 (15.1, 107.5)	37.4 (15.1, 82.7)	0.690
PCT, μg/L, median (IQR)	0.2 (0.1, 0.5)	0.2 (0.1, 0.5)	0.700
ALB, g/L, median (IQR)	36.6 (35.2, 38.8)	37.3 (35.2, 40.3)	0.464
TBIL, μmol/L, median (IQR)	14.1 (11.5, 20.6)	17.0 (12.0, 24.7)	0.188

Table 4 shows the median of maximum value for each parameter during hospital stay. BMES denotes balanced multielectrolyte solutions, NS denotes normal saline.

**Table 5:** Other secondary outcomes.

Outcomes	BMES (*n* = 30)	NS (*n* = 30)	*p*
Mechanical ventilation, hr., median (IQR)	0 (0, 122)	4 (0, 289)	0.073
ICU length of stay, d, median (IQR)	9 (6, 14)	12 (6, 20)	0.082
Hospital length of stay, d, median (IQR)	16 (11, 17)	15 (12, 22)	0.340
Hospitalization costs, 10,000¥, median (IQR)	11.5 (9.5, 19.0)	14.4 (11.0, 22.9)	0.280
GOS score at ICU discharge, median (IQR)	3 (2, 4)	3 (2, 4)	0.807
Incidence of DCI, n (%)	5 (17)	9 (30)	0.222

BMES denotes balanced multielectrolyte solutions, NS denotes normal saline, IQR denotes interquartile range, GOS denotes glasgow outcome scale, DCI delayed cerebral ischemia.

**Figure 4:**
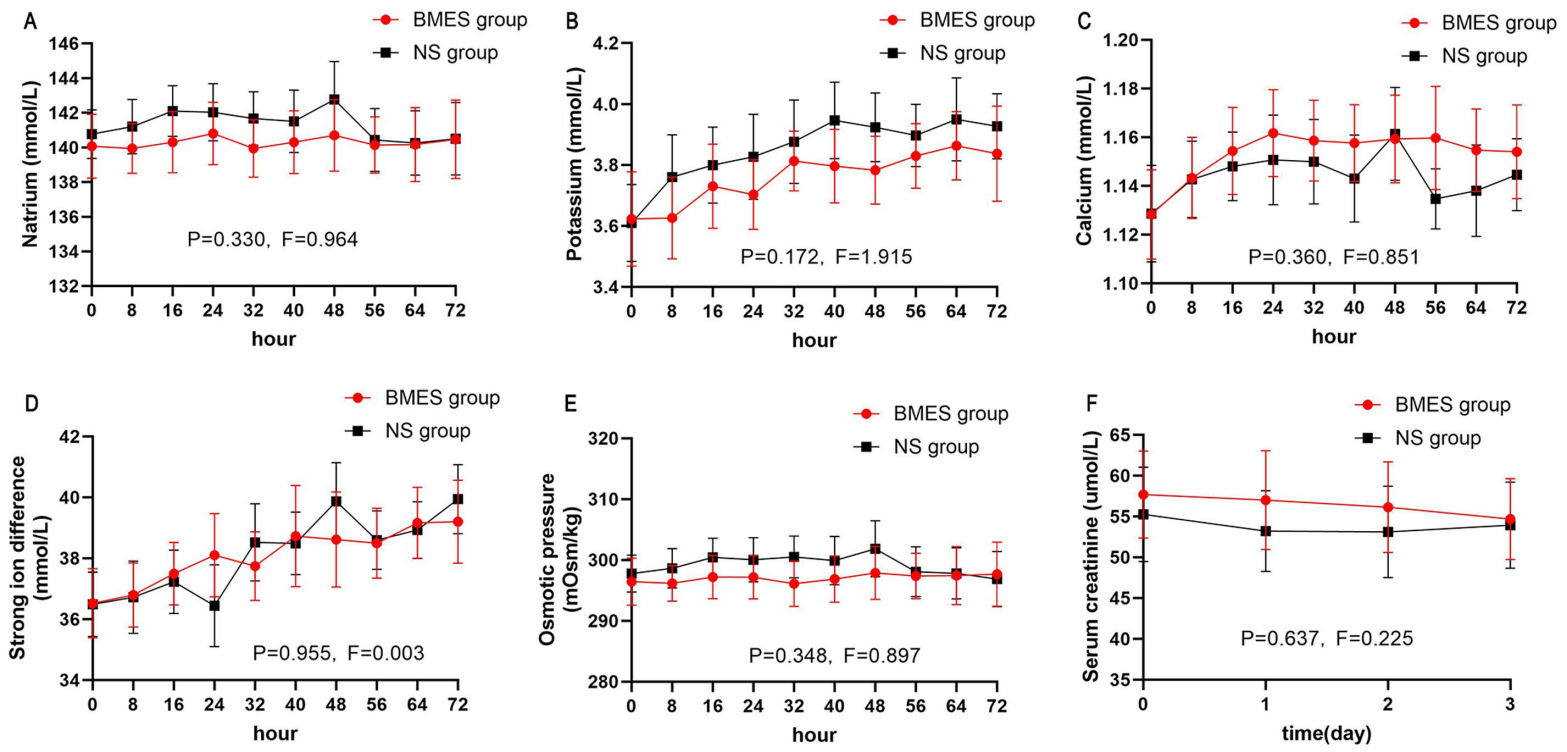
Blood biochemical parameters. The mean and 95% CI of serum natrium **(A)**, potassium **(B)**, calcium **(C)**, strong iron difference **(D)**, osmotic pressure **(E)**, serum creatinine **(F)** during the first 72 hours of treatment period are shown. Strong iron difference is calculated by: natrium + potassium – chloride. Osmotic pressure is calculated by: 2 * (natrium + potassium) + glucose.

**Figure 5:**
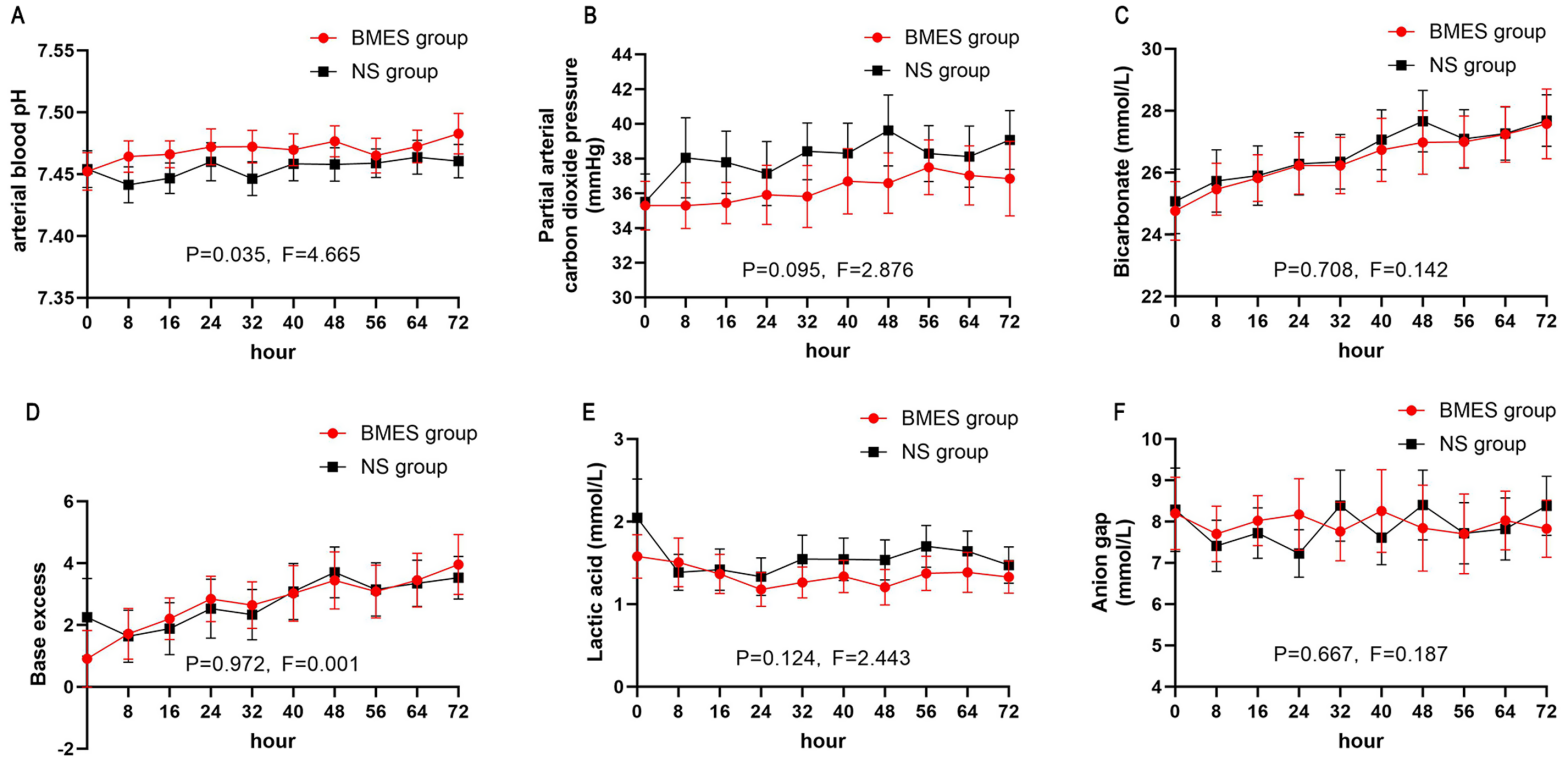
Acid-base balance. The mean and 95% CI of serum pH **(A)**, arterial partial pressure of carbon dioxide **(B)**, bicarbonate **(C)**, base excess **(D)**, arterial lactic acid **(E)**, anion gap **(F)** during the first 72 hours of treatment period are shown. Anion gap is calculated by: natrium - potassium - bicarbonate.

Other blood biochemical parameters such as serum sodium, potassium, calcium, BUN, and creatinine concentration did not differ between groups during the first 3 days after randomization (Figure 4; Supplementary Figure S1). In terms of arterial blood gas parameters during treatment, patients in the BMES group had a higher arterial pH (*F* = 4.664, *p* = 0.035), while other parameters were comparable between groups (Figure 5). Adverse events were reported in 2 of 30 (6.7%) patients receiving BMES versus 2 of 30 (6.7%) patients in patients receiving NS. The only adverse event was severe electrolyte disorder. No other adverse event was found.

## 4 Discussion

In this single-center, single-blinded, randomized, controlled pilot trial, we compared the use of intravenous BMES to NS for fluid therapy in patients with aSAH. We found that treatment with BMES did not result in a lower risk of hyperchloremia during the first 3 days after randomization as well as hyperchloric acidosis, acute kidney injury, inflammatory parameters, ICP, mechanical ventilation, hospital length of stay or GOS score at ICU discharge.

The effect of balanced crystalloid solutions on prognosis in patients with aSAH is still unknown. The current guideline recommends clinicians to choose according to their preferences (18). In 2018, SMART study (13) showed an attractive reduction in 30 days major adverse kidney event (MAKE30) in patients treated with balanced crystalloids compared to saline, and a lower incidence of hyperchloremia in five ICUs. There was an obvious difference in the subgroup analysis of neurocritical care unit whose MAKE30 was lower in the balanced crystalloids group (8.1%) than in the saline group (10.2%). Studies of neurological patients also showed a decrease in hyperchloremia and hyperchloraemic acidosis (19) as well as hyper-osmolality (20). This does not consistent with the findings of our study.

However, in our study, patients assigned to receive BMES appeared to have a lower prevalence of hyperchloremia and hyperchloric acidosis on trial day 1 and day 2. Also, the gap of serum chloride concentration was widened from 0.8 mmol/L (0 h) to 3.2 mmol/L (24 h) on the first 24 h after randomization, but then narrowed. We suspect that the occurrence of hyperchloremia may resulted in changes in clinical practice, such as more usage of glucose solution instead of saline when both solutions were appropriate for dissolving drugs. While some of these time points showed statistically significant differences, it should be interpreted cautiously due to multiple comparisons across time points. Aside from the limited sample size, the lack of significance for the primary endpoint may be attributed to the fact that a certain amount of proportion of the study subjects (11 in the study group and 12 in the controls) were lost to follow-up or withdrew their informed consent.

Although no statistical difference was found for the primary endpoint, we did find a numerical trend toward improvements in hyperchloremia incidence within 72 h of randomization. Also, the arterial pH was lower in the NS group during the first three days after randomization, indicating a potential risk of acidosis, which was consistent with the previous trial showing that balanced solution can reduce the incidence of hyperchloraemia acidosis and improve electrolyte balance in patients with brain injury (19, 21).

The BMES group had a numerically lower median SOFA score, which could reflect slightly less severe illness at baseline. Although the difference did not reach statistical significance (*p* = 0.063). However, as a pilot RCT, the primary objective of this study was to offer safety and feasibility evidence for the design of subsequent large trials. Our findings shows that BMES did not increase the incidence of hyponatremia, low osmolality or intracranial hypertension, which is a major concern in the administration of BMES in SAH patients. As a result, we plan to conduct a multicenter randomize controlled trial in the near future.

This study has several limitations. First of all, the study contains a relatively low sample size of 30 per group. Which was not powerful enough to detect a difference in the incidence of hyperchloremia within 72 h of randomization. Second, the study is conducted in one single center that limits its generalizability. In addition, the allowance of non-study intravenous fluids outside the standardized protocol may have contributed to inter-individual variability in electrolyte balance. Future trials should implement stricter protocols to control all sources of intravenous fluids, ensuring tighter adherence to the assigned intervention.

## 5 Conclusion

In this randomized trial involving patients with aSAH, BMES resulted in no difference in the incidence of hyperchloremia within 72 h of randomization comparing to NS. Our study indicated that using BMES could potentially be more effective at controlling serum chloride levels compared to NS, a finding that requires further investigation. We are in the process of organizing a larger sample size, multicenter, randomized controlled trial to gather sufficient evidence to support this potential benefit.

## Data Availability

The raw data supporting the conclusions of this article will be made available by the authors, without undue reservation.

## References

[1] Etminan N Chang HS Hackenberg K de Rooij NK Vergouwen MDI Rinkel GJE . Worldwide incidence of aneurysmal subarachnoid hemorrhage according to region, time period, blood pressure, and smoking prevalence in the population: a systematic review and Meta-analysis. JAMA Neurol. (2019) 76:588–97. doi: 10.1001/jamaneurol.2019.0006, PMID: 30659573 PMC6515606

[2] Mackey J Khoury JC Alwell K Moomaw CJ Kissela BM Flaherty ML . Stable incidence but declining case-fatality rates of subarachnoid hemorrhage in a population. Neurology. (2016) 87:2192–7. doi: 10.1212/WNL.0000000000003353, PMID: 27770074 PMC5123555

[3] Schatlo B Fung C Stienen MN Fathi AR Fandino J Smoll NR . Incidence and outcome of aneurysmal subarachnoid hemorrhage: the Swiss study on subarachnoid hemorrhage (Swiss SOS). Stroke. (2021) 52:344–7. doi: 10.1161/STROKEAHA.120.029538, PMID: 33272133

[4] Hoh BL Ko NU Amin-Hanjani S Chou S-Y Cruz-Flores S Dangayach NS . 2023 guideline for the Management of Patients with Aneurysmal Subarachnoid Hemorrhage: a guideline from the American Heart Association/American Stroke Association. Stroke. (2023) 54:e314–70. doi: 10.1161/STR.0000000000000436, PMID: 37212182

[5] Mapa B Taylor BE Appelboom G Bruce EM Claassen J Connolly ES Jr. Impact of hyponatremia on morbidity, mortality, and complications after aneurysmal subarachnoid hemorrhage: a systematic review. World Neurosurg. (2016) 85:305–14. doi: 10.1016/j.wneu.2015.08.054, PMID: 26361321

[6] Zheng B Qiu Y Jin H Wang L Chen X Shi C . A predictive value of hyponatremia for poor outcome and cerebral infarction in high-grade aneurysmal subarachnoid haemorrhage patients. J Neurol Neurosurg Psychiatry. (2011) 82:213–7. doi: 10.1136/jnnp.2009.180349, PMID: 20667862

[7] Qureshi AI Suri MF Sung GY Straw RN Yahia AM Saad M . Prognostic significance of hypernatremia and hyponatremia among patients with aneurysmal subarachnoid hemorrhage. Neurosurgery. (2002) 50:749. doi: 10.1097/00006123-200204000-00012, PMID: 11904025

[8] Wu BU Hwang JQ Gardner TH Repas K Delee R Yu S . Lactated ringer's solution reduces systemic inflammation compared with saline in patients with acute pancreatitis. Clin Gastroenterol Hepatol. (2011) 9:710–7.e1. doi: 10.1016/j.cgh.2011.04.026, PMID: 21645639

[9] Chowdhury AH Cox EF Francis ST Lobo DN. A randomized, controlled, double-blind crossover study on the effects of 2-L infusions of 0.9% saline and plasma-lyte® 148 on renal blood flow velocity and renal cortical tissue perfusion in healthy volunteers. Ann Surg. (2012) 256:18–24. doi: 10.1097/SLA.0b013e318256be72, PMID: 22580944

[10] Riha HM Erdman MJ Vandigo JE Kimmons LA Goyal N Davidson KE . Impact of moderate Hyperchloremia on clinical outcomes in intracerebral hemorrhage patients treated with continuous infusion hypertonic saline: a pilot study. Crit Care Med. (2017) 45:e947–53. doi: 10.1097/CCM.0000000000002522, PMID: 28538442

[11] Young P Bailey M Beasley R Henderson S Mackle D McArthur C . Effect of a buffered crystalloid solution vs saline on acute kidney injury among patients in the intensive care unit: the SPLIT randomized clinical trial. JAMA. (2015) 314:1701–10. doi: 10.1001/jama.2015.12334, PMID: 26444692

[12] Yunos NM Bellomo R Hegarty C Story D Ho L Bailey M. Association between a chloride-liberal vs chloride-restrictive intravenous fluid administration strategy and kidney injury in critically ill adults. JAMA. (2012) 308:1566–72. doi: 10.1001/jama.2012.13356, PMID: 23073953

[13] Semler MW Self WH Wanderer JP Ehrenfeld JM Wang L Byrne DW . Balanced crystalloids versus saline in critically ill adults. N Engl J Med. (2018) 378:829–39. doi: 10.1056/NEJMoa1711584, PMID: 29485925 PMC5846085

[14] Finfer S Micallef S Hammond N Navarra L Bellomo R Billot L . Balanced multielectrolyte solution versus saline in critically ill adults. N Engl J Med. (2022) 386:815–26. doi: 10.1056/NEJMoa2114464, PMID: 35041780

[15] Zampieri FG Machado FR Biondi RS Freitas FGR Veiga VC Figueiredo RC . Effect of intravenous fluid treatment with a balanced solution vs 0.9% saline solution on mortality in critically ill patients: the BaSICS randomized clinical trial. JAMA. (2021) 326:1–12. doi: 10.1001/jama.2021.11684, PMID: 34375394 PMC8356144

[16] Semler MW Wanderer JP Ehrenfeld JM Stollings JL Self WH Siew ED . Balanced crystalloids versus saline in the intensive care unit. The SALT randomized trial. Am J Respir Crit Care Med. (2017) 195:1362–72. doi: 10.1164/rccm.201607-1345OC, PMID: 27749094 PMC5443900

[17] Khwaja A. KDIGO clinical practice guidelines for acute kidney injury. Nephron Clin Pract. (2012) 120:c179–84. doi: 10.1159/000339789, PMID: 22890468

[18] Oddo M Poole D Helbok R Meyfroidt G Stocchetti N Bouzat P . Fluid therapy in neurointensive care patients: ESICM consensus and clinical practice recommendations. Intensive Care Med. (2018) 44:449–63. doi: 10.1007/s00134-018-5086-z, PMID: 29500701

[19] Roquilly A Loutrel O Cinotti R Rosenczweig E Flet L Mahe PJ . Balanced versus chloride-rich solutions for fluid resuscitation in brain-injured patients: a randomised double-blind pilot study. Crit Care. (2013) 17:R77. doi: 10.1186/cc12686, PMID: 23601796 PMC4057192

[20] Lehmann L Bendel S Uehlinger DE Takala J Schafer M Reinert M . Randomized, double-blind trial of the effect of fluid composition on electrolyte, acid-base, and fluid homeostasis in patients early after subarachnoid hemorrhage. Neurocrit Care. (2013) 18:5–12. doi: 10.1007/s12028-012-9764-3, PMID: 22872427

[21] Hassan MH Hassan W Zaini RHM Shukeri W Abidin HZ Eu CS. Balanced fluid versus saline-based fluid in post-operative severe traumatic brain injury patients: acid-base and electrolytes assessment. Malays J Med Sci. (2017) 24:83–93. doi: 10.21315/mjms2017.24.5.9, PMID: 29386975 PMC5772818

