# Supplementary material for: Balanced multi-electrolyte solution versus normal saline for fluid therapy in aneurysmal subarachnoid hemorrhage: an influence on fluid and electrolytes balance and outcome
Source: Front Med (Lausanne). 2025 Nov 26;12:1708812. doi: 10.3389/fmed.2025.1708812 (PMC12689972; doi:10.3389/fmed.2025.1708812)
Supplement: Supplementary file 1 [file Data_Sheet_1.docx]

**Figure S1. Blood urea nitrogen during the treatment period.** BMES denotes balanced multielectrolyte solutions, NS denotes normal saline.


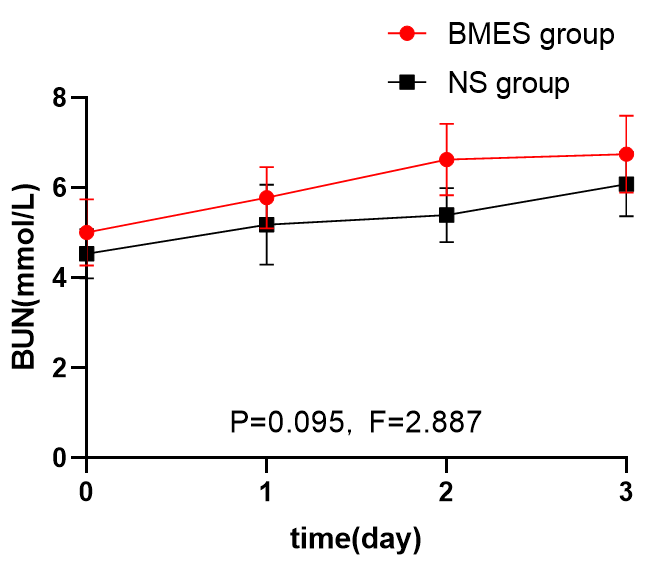


**Table S1. Ingredients of study fluids.**

|  | Sterofundin | | Normal Saline |
| --- | --- | --- | --- |
| Natrium, mmol/L | 145 | 154 | |
| Potassium, mmol/L | 4 | 0 | |
| Calcium, mmol/L | 2.5 | 0 | |
| Magnesium, mmol/L | 1 | 0 | |
| Chlorine, mmol/L | 127 | 154 | |
| Acetic acid, mmol/L | 24 | 0 | |
| Malic acid, mmol/L | 5 | 0 | |
| Glucose, mmol/L | 0 | 0 | |
| pH | 5.1-5.9 | 5.5 | |
| Osmolality, mOsm/L | 309 | 308 | |

**Table S2. Baseline electrolyte and acid-base parameters.**

|  | BMES group（n=30） | NS group（n=30） | p |
| --- | --- | --- | --- |
| Cr, umol/L, mean (SD) | 57.7 (14.3) | 55.3 (15.5) | 0.531 |
| BUN, mmol/L, mean (SD) | 5.0 (2.0) | 4.5 (1.5) | 0.293 |
| Cl, mmol/L, mean (SD) | 107.2 (3.9) | 108.0 (3.8) | 0.432 |
| Na, mmol/L, mean (SD) | 140.0 (4.9) | 140.8 (3.8) | 0.539 |
| K, mmol/L, mean (SD) | 3.6 (0.4) | 3.3 (0.3) | 0.892 |
| Ca, mmol/L, mean (SD) | 1.13 (0.05) | 1.13 (0.05) | 0.980 |
| Lac, mmol/L, mean (SD) | 1.6 (0.7) | 2.0 (1.3) | 0.081 |
| HCO_3_, mmol/L, mean (SD) | 24.7 (2.4) | 25.1 (2.8) | 0.621 |
| ph, mean (SD) | 7.45 (0.04) | 7.45 (0.04) | 0.871 |
| PCO_2_, mmHg, mean (SD) | 35.3 (3.7) | 35.5 (4.3) | 0.841 |
| Osm, mOsm/kg, mean (SD) | 296.4 (10.4) | 297.8 (8.2) | 0.581 |
| BE, mmol/L, mean (SD) | 0.9 (2.5) | 2.3 (3.4) | 0.082 |
| AG, mmol/L, mean (SD) | 8.2 (2.4) | 7.9 (3.2) | 0..720 |
| SID, mmol/L, mean (SD) | 36.5 (3.0) | 36.5 (2.8) | 0.965 |

BMES denotes balanced multielectrolyte solutions, NS denotes normal saline. Cr denotes serum creatinine. BUN denotes blood urea nitrogen. Cl denotes chlorine. Na denotes natrium. K denotes potassium. Ca denotes calcium. Lac denotes lactic acid. HCO_3_ denotes bicarbonate. PCO_2_ denotes arterial partial pressure of carbon dioxide. Osm denotes serum osmolality calculated by 2 * (Na + K) + Glu. BE denotes base excess. AG denotes anion gap calculated by Na - Cl - HCO3. SID denotes strong ion difference calculated by Na + K - Cl.

**Table S3. Fluid intakes during intervention period.**

|  | BMES group (n=30) | NS group (n=30) | *p* |
| --- | --- | --- | --- |
| Fluid imput, ml, median (IQR) |  |  |  |
| 0 to 24h | 3300 (2800, 4065) | 3550 (3275, 4025) | 0.420 |
| 24 to 48h | 3550 (3875, 4000 | 3600 (3400, 3925) | 0.293 |
| 48 to 72h | 3420 (3000, 4050) | 3750 (3388, 4300 | 0.232 |
| 0 to 72h | 10225 (9034, 12525) | 11039 (10088, 12125) | 0.137 |
| Fluid output, ml, median (IQR) |  |  |  |
| 0 to 24h | 3435 (2778, 3960) | 3345 (2840, 3972) | 0.795 |
| 24 to 48h | 3235 (3695, 3918 | 3450 (3230, 3880) | 0.208 |
| 48 to 72h | 3415 (2867, 3875) | 3504 (3040, 3981) | 0.280 |
| 0 to 72h | 9525 (8825, 11073) | 10330 (9330, 11653) | 0.220 |
| BMES, ml, median (IQR) |  |  |  |
| 0 to 24h | 1200 (1000, 2000) | 0 (0, 0) | ＜0.001 |
| 24 to 48h | 1500 (1000, 1500） | 0 (0, 0) | ＜0.001 |
| 48 to 72h | 1000 (1000, 1500) | 0 (0, 0) | ＜0.001 |
| 0 to 72h | 4000 (3000, 4625) | 0 (0, 0) | ＜0.001 |
| NS, ml, median (IQR) |  |  |  |
| 0 to 24h | 0 (0, 500) | 1850 (1000, 2000) | ＜0.001 |
| 24 to 48h | 0 (0, 500) | 1500 (1000, 1850) | ＜0.001 |
| 48 to 72h | 0 (0, 500) | 1300 (1000, 2000) | ＜0.001 |
| 0 to 72h | 500 (0, 1275) | 4150 (3425, 5500) | ＜0.001 |
| 10% sodium chloride, ml, median (IQR) |  |  |  |
| 0 to 24h | 0 (0, 0) | 0 (0, 8) | 0.246 |
| 24 to 48h | 0 (0, 0) | 0 (0, 0) | 0.560 |
| 48 to 72h | 0 (0, 0) | 0 (0, 20) | 0.107 |
| 0 to 72h | 0 (0, 0) | 0 (0, 45) | 0.121 |
| 3% sodium chloride, ml, median (IQR) |  |  |  |
| 0 to 24h | 0 (0, 0) | 0 (0, 0) | 0.321 |
| 24 to 48h | 0 (0, 0) | 0 (0, 0) | 0.321 |
| 48 to 72h | 0 (0, 0) | 0 (0, 0) | 0.321 |
| 0 to 72h | 0 (0, 0) | 0 (0, 0) | 0.317 |
| Mannitol, ml, median (IQR) |  |  |  |
| 0 to 24h | 375 (0, 750) | 438 (250, 750) | 0.228 |
| 24 to 48h | 375 (0, 750) | 625 (375, 750) | 0.289 |
| 48 to 72h | 375 (0, 750) | 750 (375, 750) | 0.195 |
| 0 to 72h | 1125 (0, 2063) | 1500 (1000, 2250) | 0.244 |
| 5% glucose solution, ml, median (IQR) |  |  |  |
| 0 to 24h | 0 (0, 0) | 0 (0, 0) | 0.937 |
| 24 to 48h | 0 (0, 0) | 0 (0, 0) | 0.656 |
| 48 to 72h | 0 (0, 0) | 0 (0, 0) | 0.350 |
| 0 to 72h | 0 (0, 0) | 0 (0, 0) | 0.671 |
| Other balanced solution***^a^***, ml, median (IQR) |  |  |  |
| 0 to 24h | 0 (0, 350) | 0 (0, 0) | 0.254 |
| 24 to 48h | 0 (0, 0) | 0 (0, 500) | 0.042 |
| 48 to 72h | 0 (0, 75) | 0 (0, 0) | 0.290 |
| 0 to 72h | 0 (0, 500) | 0 (0, 625) | 0.766 |
| Artificial colloidal, ml, median (IQR) |  |  |  |
| 0 to 24h | 0 (0, 0) | 0 (0, 0) | 0.769 |
| 24 to 48h | 0 (0, 0) | 0 (0, 0) | 0.943 |
| 48 to 72h | 0 (0, 0) | 0 (0, 0) | 0.167 |
| 0 to 72h | 0 (0, 0) | 0 (0, 125) | 0.477 |
| 20% Albumin, ml, median (IQR) |  |  |  |
| 0 to 24h | 0 (0, 0) | 0 (0, 0) | 0.499 |
| 24 to 48h | 0 (0, 0) | 0 (0, 0) | 0.342 |
| 48 to 72h | 0 (0, 0) | 0 (0, 0) | 0.549 |
| 0 to 72h | 0 (0, 0) | 0 (0, 0) | 0.753 |
| Diet intake***^b^***, ml, median (IQR) |  |  |  |
| 0 to 24h | 950 (500, 1190) | 1020 (603, 1500) | 0.561 |
| 24 to 48h | 1065 (738, 1540) | 1050 (593, 1530) | 0.858 |
| 48 to 72h | 1020 (840, 1505) | 1270 (975, 1530) | 0.522 |
| 0 to 72h | 2935 (2333, 4180) | 3150 (2403, 4500) | 0.641 |
| Parenteral nutrition, ml, median (IQR) |  |  |  |
| 0 to 24h | 0 (0, 0) | 0 (0, 0) | 0.848 |
| 24 to 48h | 0 (0, 0) | 0 (0, 0) | 0.517 |
| 48 to 72h | 0 (0, 0) | 0 (0, 0) | 0.572 |
| 0 to 72h | 0 (0, 400) | 0 (0, 550) | 0.831 |
| Compatibility solution (NS)***^c^***, ml, median (IQR) |  |  |  |
| 0 to 24h | 400 (198, 651) | 475 (300, 693) | 0.429 |
| 24 to 48h | 425 (284, 686) | 575 (350, 756) | 0.219 |
| 48 to 72h | 450 (338, 738) | 570 (400, 798) | 0.228 |
| 0 to 72h | 1348 (850, 2009) | 1525 (1088, 2209) | 0.198 |
| Compatibility solution (5%Glucose)***^c^***, ml, median (IQR) |  |  |  |
| 0 to 24h | 0 (0, 0) | 0 (0, 100) | 0.321 |
| 24 to 48h | 0 (0, 0) | 0 (0, 100) | 0.191 |
| 48 to 72h | 0 (0, 0) | 0 (0, 100) | 0.130 |
| 0 to 72h | 0 (0, 0) | 0 (0, 263) | 0.080 |
| 15% Potassium chloride solution, ml, median (IQR) |  |  |  |
| 0 to 24h | 10 (0, 30) | 40 (24, 50) | ＜0.001 |
| 24 to 48h | 10 (0, 27) | 30 (20, 45) | ＜0.001 |
| 48 to 72h | 10 (0, 25) | 24 (20, 33) | 0.001 |
| 0 to 72h | 30 (15, 86) | 103 (70, 120) | ＜0.001 |
| Other drugs***^d^***, ml, median (IQR) |  |  |  |
| 0 to 24h | 11 (0, 70) | 52 (20, 100) | 0.056 |
| 24 to 48h | 50 (0, 103) | 50 (11, 100) | 0.910 |
| 48 to 72h | 37 (0, 103) | 50 (25, 65) | 0.867 |
| 0 to 72h | 140 (11, 225) | 157 (102, 227) | 0.249 |

a, Other balancaed solutions included lactated Ringer's solution and compound electrolytes and glucose jnjection MG3; b, The diet intakes includes nasal-fed intestinal nutrient solution and mouth-feeding liquid; c, The compatibility solution refers to the liquid used to dissolve and dilute drugs; d, Other drugs include various intravenous drugs that do not need to be dissolved, such as propofol and nicodipine, etc.. BMES denotes balanced multielectrolyte solutions, NS denotes normal saline, IQR denotes interquartile range.

**Table S4. Chloride concentration during 72 hours study period.**

|  | 0h | 8h | 16h | 24h | 32h | 40h | 48h | 56h | 64h | 72h |
| --- | --- | --- | --- | --- | --- | --- | --- | --- | --- | --- |
| BMES, mmol/L,  mean (SD) | 107.2 (3.9) | 106.8 (4.4) | 106.5 (4.7) | 106.4 (5.3) | 106.0 (5.3) | 105.4 (6.0) | 105.9 (6.0) | 105.5 (5.3) | 104.9 (5.8) | 105.1 (5.9) |
| NS, mmol/L,  mean (SD) | 108.0 (3.8) | 108.4 (3.3) | 109.0 (3.5) | 109.6 (4.6) | 107.2 (4.4) | 107.2 (4.5) | 107.0 (4.5) | 105.8 (4.0) | 105.4 (4.4) | 104.7 (5.5) |
| p | 0.432 | 0.115 | 0.029 | 0.017 | 0.331 | 0.182 | 0.416 | 0.813 | 0.704 | 0.801 |

BMES denotes balanced multielectrolyte solutions, NS denotes normal saline.
